# Rapid Insulin-Mediated Increase in Microvascular Glycocalyx Accessibility in Skeletal Muscle May Contribute to Insulin-Mediated Glucose Disposal in Rats

**DOI:** 10.1371/journal.pone.0055399

**Published:** 2013-01-31

**Authors:** Bart J. M. Eskens, Hans L. Mooij, Jack P. M. Cleutjens, Jozef M. A. Roos, Johanna E. Cobelens, Hans Vink, Jurgen W. G. E. VanTeeffelen

**Affiliations:** 1 Department of Physiology, Cardiovascular Research Institute Maastricht, Maastricht University, Maastricht, The Netherlands; 2 Department of Vascular Medicine, Academic Medical Center, University of Amsterdam, Amsterdam, The Netherlands; 3 Department of Pathology, Cardiovascular Research Institute Maastricht, Maastricht University, Maastricht, The Netherlands; Universidade Federal do Rio de Janeiro (UFRJ), Brazil

## Abstract

It has been demonstrated that insulin-mediated recruitment of microvascular blood volume is associated with insulin sensitivity. We hypothesize that insulin rapidly stimulates penetration of red blood cells (RBC) and plasma into the glycocalyx and thereby promotes insulin-mediated glucose uptake by increasing intracapillary blood volume. Experiments were performed in rats; the role of the glycocalyx was assessed by enzymatic degradation using a bolus of hyaluronidase. First, the effect of insulin on glycocalyx accessibility was assessed by measuring the depth of penetration of RBCs into the glycocalyx in microvessels of the gastrocnemius muscle with Sidestream Dark-field imaging. Secondly, peripheral insulin sensitivity was determined using intravenous insulin tolerance tests (IVITT). In addition, in a smaller set of experiments, intravital microscopy of capillary hemodynamics in cremaster muscle and histological analysis of the distribution of fluorescently labeled 40 kDa dextrans (D40) in hindlimb muscle was used to evaluate insulin-mediated increases in capillary blood volume. Insulin increased glycocalyx penetration of RBCs by 0.34±0.44 µm (P<0.05) within 10 minutes, and this effect of insulin was greatly impaired in hyaluronidase treated rats. Further, hyaluronidase treated rats showed a 35±25% reduction in whole-body insulin-mediated glucose disposal compared to control rats. Insulin-mediated increases in capillary blood volume were reflected by a rapid increase in capillary tube hematocrit from 21.1±10.1% to 29.0±9.8% (P<0.05), and an increase in D40 intensity in individual capillaries of 134±138% compared to baseline at the end of the IVITT. These effects of insulin were virtually abolished in hyaluronidase treated animals. In conclusion, insulin rapidly increases glycocalyx accessibility for circulating blood in muscle, and this is associated with an increased blood volume in individual capillaries. Hyaluronidase treatment of the glycocalyx abolishes the effects of insulin on capillary blood volume and impairs insulin-mediated glucose disposal.

## Introduction

After insulin has been secreted by the pancreas into the bloodstream, it is distributed by the microcirculation towards the capillaries where it has to cross the endothelium to bind to insulin receptors on target tissues, such as skeletal muscle. There is increasing evidence that both insulin delivery by the blood and the subsequent exchange from capillaries to the tissue may be rate limiting steps for the metabolic actions of insulin [1], [2], [3]. In skeletal muscle microcirculation, insulin has been indicated to rapidly (within 10–15 minutes) recruit capillary blood volume [4], [5], an effect which occurs before an increase of total blood flow, and which appears to be important for insulin’s metabolic actions [5]. While there is substantial experimental evidence for this link between the ability of insulin to increase capillary blood volume and its ability to efficiently dispose glucose from the circulation [5], [6], the mechanism by which insulin mediates the increase in capillary blood volume in muscle is not well understood at the moment [7]. While traditionally the increase in capillary blood volume has been explained by an increase in number of perfused capillaries, our previous studies suggest that agonists are also able to increase functionally perfused capillary blood volume within individual microvessels by modulating the barrier properties of the endothelial glycocalyx [8], [9], [10]. The endothelial glycocalyx is the 0.5–1.0 µm thick gel-like layer on the luminal side of the vascular endothelial cells. It consists of a mesh of polysaccharide structures and absorbed plasma proteins and water [11], [12], and has the last decade been indicated to have an important role in protection of the vessel wall, as well as in regulation of microvascular perfusion and nutrient exchange [9], [13]. Intravital microscopy studies in rodent cremaster muscle indicate that under control conditions the glycocalyx is to a large extent inaccessible for red blood cells (RBCs) and plasma macromolecules [14], [15], but that its accessibility can be rapidly increased in the presence of the agonists adenosine, bradykinin and sodium nitroprusside, resulting in a robust increase in capillary tube hematocrit [8], [10], [16]. Conversely, glycocalyx degradation during chronic hyperlipidemic conditions or acute enzymatic treatment has been associated with an impaired ability for these agonists to increase capillary blood volume [10], [17].

The aim of the current study was to determine whether insulin also has the ability to rapidly increase blood volume in individual capillaries in muscle by modulation of the glycocalyx, and if this mechanism is important for insulin-mediated glucose disposal. In rats, we measured insulin effects on microvascular glycocalyx barrier properties and capillary blood volume in muscle, and assessed insulin sensitivity by an intravenous insulin tolerance test. The role of the glycocalyx was evaluated by acute enzymatic degradation with hyaluronidase [18], [19]. Our results indicate that insulin, by rapidly increasing glycocalyx accessibility for circulating blood, is able to augment blood volume within individual capillaries in skeletal muscle, and this effect is indicated to contribute to insulin-mediated glucose disposal.

## Methods

### Animals and General Instrumentation

The experimental protocols were approved by the Animal Ethics Care and Use committee of Maastricht University (AEC protocol numbers: 2007–167 and 2009–015). Experiments were performed in male Wistar rats (350–400 gram; n = 42) that received standard chow and water ad libitum. After arrival from the external supplier (Harlan, Horst, The Netherlands) the animals were housed at the animal facility of Maastricht University and allowed to acclimatize for at least one week before the experiments. At the day of the experiment, after an overnight fast (10–12 h), rats received the narcotic analgesic buprenorphine (*Temgesic 0.03 mg/mL;* Schering-Plough*) at 0.1 mg/kg subcutaneously*, while 30–45 minutes later the animals were put under isoflurane anesthesia (2%). Their femoral vein was cannulated with a polyurethane catheter (1.02 mm outer diameter (OD); 0.61 mm inner diameter (ID), Charles River Wiga GmbH, Sulzfeld, Germany), and either a bolus of 1 ml saline or 1 ml hyaluronidase (500 U/ml) was infused. One hour later baseline glucose (Gluc_0_) and baseline insulin (Ins_0_) were measured in each animal, and as a measure for baseline insulin sensitivity the HOMA-IR was calculated ((Ins_0_×Gluc_0_)/405). Insulin infusion started 60–90 min after bolus injection in the different protocols described below. The choice for a 1–1.5 hour incubation period was based on previous reports of a time-dependent effect of bolus administration of hyaluronidase on impairment of glycocalyx exclusion properties [18], [19]. At the end of the experiments rats were sacrificed by exsanguination while under anesthesia.

### Experimental Protocols

Table 1 and 2 depicts the different protocols used in the current study. Two main protocols were used. In the first protocol (Table 1), it was studied whether glycocalyx barrier properties were rapidly impaired upon insulin infusion, and in the second (Table 2) it was studied whether enzymatic glycocalyx degradation was associated with an impaired insulin sensitivity. To evaluate whether the effects observed in these two studies were associated with changes in perfused capillary blood volume, for each protocol additional experiments were performed in a smaller group of animals (named protocol 1a and 2a, respectively).

**Table 1:** Overview of the number of animals used in protocol 1; in addition, the number of vessels (SDF and IVM imaging) that were analyzed is given.

Protocol	Method	Control rats				Hyaluronidase treated rats			
		Baseline		Insulin		Baseline		Insulin	
		Number ofrats	Number of vessels	Number ofrats	Number of vessels	Number ofrats	Number of vessels	Number ofrats	Number of vessels
**1) Does insulin affect microvascular glycocalyx barrier properties?**	SDF imaging	6	54	6	54	7	72	7	72
1a) Is this associated with an increase in capillary RBC volume?	IVM	3	13	3	14	2	9	2	7

In these protocols data were obtained in the same rats during baseline conditions and during insulin administration. In protocol 1 same vessels within one animal were monitored during baseline and insulin, allowing paired comparison; in protocol 1a, different vessels were monitored during both conditions (unpaired comparison).

**Table 2:** Overview of the number of animals used in protocol 2; in addition, the number of slides (D40 distribution) that were analyzed is given.

Protocol	Method	control rats				hyaluronidase treated rats			
		Baseline		Insulin		Baseline		Insulin	
		Number ofrats	Number of slides	Number ofrats	Number of slides	Number ofrats	Number of slides	Number ofrats	Number of slides
**2) Does glycocalyx degradation affect insulin sensitivity?**	IVITT	6	–	6	–	7	–	7	–
2a) Is this reflected by an impaired capillary plasma volume increase?	Histology	4	18	3^*^	13	4	17	3^*^	11

In protocol 2 data were obtained in the same rats during baseline conditions and during insulin administration. Protocol 2a involved 4 different groups of rats; for the insulin condition hindlimb muscles from a subset of rats that underwent the IVITT in protocol 2 were used (indicated by *). Furthermore, additional rats (n = 3) were used in this protocol to measure the contribution of autofluorescence (see Methods).

#### 1) Rapid effect of insulin on glycocalyx barrier properties in hindlimb muscle

To determine the potency of insulin to rapidly modulate microvascular glycocalyx barrier properties, rats received a constant infusion of insulin (6 mU/min/kg), somatostatin (0.8 µg/kg/min) and glucose (12 mg/kg/min) for 30 minutes. Somatostatin was used to block endogenous insulin and glucagon secretion. The co-infusion of glucose enabled blood glucose levels to be maintained above 75 mg/dl, as checked by regular blood glucose measurements (Ascensia Contour). To measure the effect of insulin on glycocalyx barrier properties, changes in RBC column width in individual microvessels were determined. Herefore, the superficial part of the gastrocnemius muscle in rats was exposed, and a bicarbonate-buffered physiological salt solution (PSS) of the following composition (mM): 131.9 NaCl, 4.7 KCl, 2.0 CaCl2, 1.2 MgSO4, 20 NaHCO3 and equilibrated with 5% CO_2_–95% N_2_ to obtain a pH of ±7.4 was used to suffuse the muscle. The microcirculation was imaged with a SDF camera, which is equipped with a 5X magnifying objective lens system-containing probe, imaging the RBCs in the tissue-embedded microcirculation using green pulsed LED ring illumination [20]. After equilibration, the microcirculation was visualized 10 times at baseline (for 30 minutes) and during the constant infusion of insulin for 30 minutes. In each animal, paired measurements of the muscle microcirculation were made by holding the SDF at the same position using a micromanipulator.

**1a) Rapid Effect of Insulin on Capillary Tube Hematocrit in Cremaster Muscle.** To test if the rapid changes (i.e., within 10 minutes) in glycocalyx barrier properties by insulin were accompanied by concomitant rapid increases in capillary blood volume, capillary tube hematocrit measurements were performed in cremaster muscle using intravital microscopy. Herefore, rats were placed in a supine position on a custom built animal platform and the right cremaster muscle was prepared as previously described for the mouse [21], [22]. Briefly, an incision was made through the skin and the muscle dissected from the surrounding connective tissue. The exposed muscle was positioned on a clear Silicon pedestal and longitudinally incised from the apex to the inguinal canal with minimal disruption of the vascular supply. After severing the deferential artery and vein, the testis and epididymis were dissected away and repositioned in the abdominal cavity. The cremaster muscle was spread radially on the pedestal and pinned at the edges. The muscle was continuously (∼5 ml min^−1^) superfused at 34°C with PSS. Following surgery, the completed preparation was transferred to the stage of an intravital microscope (Leica Microsystems, Wetzlar, Germany), coupled to a CCD video camera (C9100; Hamamatsu, Hamamatsu City, Japan). Microvessels were observed using bright-field (100 W Hg lamp) microscopy (condensor: numerical aperture (NA): 0.6; salt water immersion objective lens (SW100, NA 1.2)). Bright-field images were made with a 435 nm band pass interference filter (blue light) in the light path. The preparation was equilibrated for 20–30 min, during which time the arteriolar network was scanned for the presence of vasomotor tone. Cremaster capillaries from different microscopic fields were randomly chosen for examination and recorded with Wasabi software (Hamamatsu) before (for 30 minutes) and during 10 minutes after start of the insulin infusion.

#### 2) Effect of enzymatic glycocalyx treatment on insulin-mediated glucose disposal

An intravenous insulin tolerance test (IVITT) was performed to measure insulin-mediated glucose disposal. To avoid hypoglycemia resulting from the insulin infusion, first a bolus of 1 g/kg glucose (0.5 g/mL) was given via the venous line and this was followed by a bolus of 1 U/kg insulin (1 U/mL) after 30 minutes. Blood glucose was measured, via tail bleeding, with a glucose meter (Ascensia contour) at t = −10 and 0 (pre) and t = 2, 4, 6, 8, 10, 15, 20 and 30 minutes after glucose infusion; then the bolus of insulin at t = 31 was given and blood glucose was further monitored at t = 33, 35, 37, 39, 41, 46, 51, and 61 minutes. Plasma insulin levels were measured with an ELISA (ALPCO Diagnostics, Salem, NH) at t = 0 (pre) and t = 2, 6, 10, 15, 30, 33, 37, 41, 46 and 61 minutes after glucose infusion; systemic hematocrit was also measured at these time points.

**2a) Effect of Enzymatic Glycocalyx Treatment on Insulin-mediated Increases in Plasma Volume in Hindlimb Capillaries.** At the end of the IVITT (t = 61 min), 0.5 ml of Texas-Red labeled 40 kDa dextrans (5 mg/ml) (D40; Invitrogen) was infused, and 3 minutes later hindlimb muscles were rapidly removed and immediately fixed in a 4% formaldehyde solution for subsequent histological analysis. Additional control experiments were performed to determine the D40 distribution in the absence of insulin, and to correct for the contribution of autofluorescence (no D40 infusion).

#### Data Analysis

##### 1) Glycocalyx barrier properties

**Figure 1:**
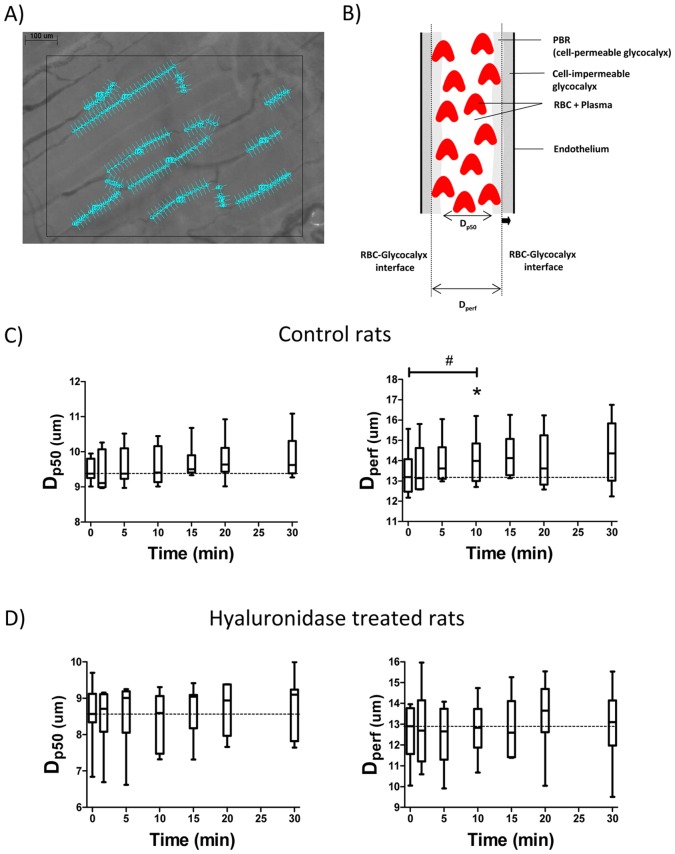
Example of a single SDF image of rat gastrocnemius microcirculation is shown in panel A. From each movie (total length: 40 frames) recorded by the SDF camera the distribution of the width of the red blood cell (RBC) column in each microvessel that fitted the criteria of the analysis (a) in focus, b) had a sufficient length and were not close to bifurcations, and c) were continuously filled with RBCs and did not contain plasma gaps) was determined along the vessel by placement of lines ± every 10 µm perpendicular to the vessel direction (as shown by numbered lines in image), and calculating the full width at half maximum of the intensity profile 10 times in front and 10 times behind each line frame by frame in all 40 consecutive frames (see methods). Per vessel, the D_P50_ and D_perf_, by linear extrapolation of the distribution of RBC column width, were determined, revealing the perfused boundary region (PBR), which includes the cell-permeable part of the glycocalyx (see Discussion, page 16). Between the PBR and the endothelium the cell-impermeable glycocalyx resides. An increase in the accessibility of this part of the glycocalyx for the flowing RBCs is associated with an outward movement of the RBC-glycocalyx interface as shown by the thick arrow in panel B. Effects of insulin on the D_p50_ and D_perf_ in control rats (n = 6) are shown in panel C and effect of insulin on the D_p50_ and D_Perf_ in hyaluronidase treated rats (n = 7) are shown in panel D. #, P<0.05 (1-way ANOVA), *, P<0.05 versus baseline (t = 0) (Tukey’s post hoc test).

Insulin-mediated changes in glycocalyx barrier properties were derived from dynamic RBC column width measurements, which were determined in all visualized microvessels that were a) in focus, b) had a sufficient length and were not close to bifurcations, and c) were continuously filled with RBCs and did not contain plasma gaps. Movies consisted of 40 consecutive frames and contained 5–15 useful microvessels per observed muscle area (950 µm by 700 µm) (Fig. 1A). In each frame, lines were placed every 10 µm perpendicular to the vessel direction along the length of each visible microvessel. Each line represented a measurement site; at each site a total of 21 parallel (every ±0.5 µm) intensity profiles was plotted (using ImageJ, National Institutes of Health, Bethesda, MD) and RBC column width (full width half maximum (FWHM)) was determined at each line for all 40 consecutive frames in a movie, revealing a total of 840 RBC column width measurements at a single measurement site (21 profiles×40 frames). The cumulative distribution of the RBC column widths for these 840 measurements was constructed and used to determine median RBC column diameter (D_P50_) at each measurement site; in addition, RBC column widths percentiles between P25 and P75 were fitted with a linear fit to determine the distance between opposite positions of the RBC-glycocalyx interface at each measurement site, which is defined as the RBC-perfused diameter (D_perf_) (Fig. 1B) [23]. For each microvessel in an experiment, D_P50_ and D_perf_ were averaged for all measurement sites along the vessel, and this was done at each time point.

**1a) Capillary Tube Hematocrit.** Capillary hemodynamics in 4–5 capillaries per muscle were monitored at baseline and during insulin infusion for 10 minutes. Capillary tube hematocrit (Hcap) was determined from the digitally recorded movies from capillary anatomical diameter (D_a_), the flux of RBCs (F) and the velocity of RBCs (V) in each recorded capillary using the formula:
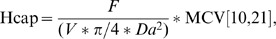



where MCV is the mean corpuscular volume in rats (±70 um^3^) [24], [25]. Using frame by frame playback RBC flux in a capillary was determined, at least 5 times per measurement, by counting the number of RBCs to pass through a reference point in 1 second. The velocity of RBCs in each capillary was determined by measuring the length of a capillary segment and dividing it by the time required for RBCs to traverse this segment [21]. Further, capillary blood flow (Qcap) was calculated using the formula:



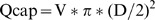



##### 2) Insulin-mediated glucose disposal

As a reflection of the insulin sensitivity during the IVITT, the decline in blood glucose between 2 and 30 minutes after insulin infusion was determined, and the glucose disposal rate (Kitt) calculated from the slope of the linear regression line of the logarithm of blood glucose against time multiplied by −100 [26].

**2a) D40 Distribution.** Muscles were paraffin-embedded and sectioned in the transverse direction. Of each muscle 5 slides were analyzed; only slides were included in which microvessels were sectioned perpendicular, as checked by the hexagonal pattern of the cross-sectioned muscle fibers and roundness of the D40/WGA-FITC stain (see below). Images (713 µm×532 µm) were captured with a Leica DFC320 digital camera (Leica, Rijswijk, Netherlands) at 20× magnification (Leica DM3000 microscope). Within a slide both diameter and total intensity of each D40 filled capillary were derived using MATLAB software (MATLAB 7.8.0). Thereto, the number of fluorescent pixels per capillary was used as a measurement of the total surface area (A) of a filled capillary, and diameter (D) calculated using the formula:
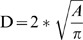



Total intensity per filled capillary was calculated by dividing the intensity of each fluorescent pixel in the vessel by the mean background intensity from the surrounding muscle tissue, and subsequent summation of all normalized pixel intensities. In the slides that were analyzed, for all four conditions, approximately 85% of all microvessels had a D40 diameter <10 µm, indicating that primarily capillaries were analyzed.

In addition, in each slide the total number of D40 perfused capillaries per muscle area was calculated using specialized morphometry software (Leica QWin V3, Cambridge, United Kingdom). For reference, structural capillary density was measured in a number of the slides as well. Hereto, slides were deparaffinized and stained using 50 µg/ml FITC-labeled lectin from triticum vulgaris (WGA-FITC; Sigma). Images were captured at the same x-y position of the slide as for the D40 image and the total number of WGA-FITC stained capillaries per muscle area was calculated also using specialized morphometry software.

#### Statistical Analysis

Statistical analysis for protocols 1 and 2 was performed on the number of animals; for protocols 1a and 2a analysis was performed on the number of vessels and number of slides, respectively. These numbers were based on power analyses (power = 0,8; α = 0.05). Thus, the study of Vincent et al. [5], showed, using contrast-enhanced ultrasound, that insulin rapidly increased microvascular blood volume by ∼50%, and we supposed that insulin would have comparable effects on capillary tube hematocrit (SD: 40%) and D40 intensity (SD: 41%), revealing that a total number of 11 vessels and 12 slides were needed, respectively, to observe significant increases by insulin. The number of animals that were used in each protocol as well as the number of vessels (SDF and IVM imaging) or slides (D40 distribution) that were analyzed are summarized in Table 1 and 2. All data are presented as means ± SD; coefficient of variation is expressed as (SD/mean×100%). The normality of the data was checked with the D'Agostino & Pearson omnibus normality test for groups with a n>6 and the Shapiro Kolmogorov-Smirnov test for groups with a n<6. If data were not normally distributed, the range (minimum to maximum) has been added. The effect of insulin on glycocalyx barrier properties measured with SDF was tested for the first 10 minutes as well as for the entire 30 minute infusion period, a 1-way ANOVA test with Tukey’s posthoc test was used if all data passed the normality test, while a Friedman test with Dunn's post-hoc multiple comparison test was used in case when groups did not pass the normality test. Statistical differences in the other experiments were tested with Student’s t-tests or Mann-Whitney U test. A value of P<0.05 was considered statistically significant.

## Results

To determine the potency of insulin to rapidly modulate microvascular glycocalyx properties and thereby increase blood volume in capillaries (Protocol 1), control and hyaluronidase treated rats received a constant infusion of insulin (6 mU/min/kg), somatostatin (0.8 µg/kg/min) and glucose (12 mg/kg/min) for 30 minutes. Baseline glucose and insulin levels were 115.0±30.4 (75.7 −198.2) mg/dl and 7.6±1.5 (5.7 −9.7) µU/ml respectively in control rats and were not different in the hyaluronidase treated rats (130.0±29.6 (82.9 −187.4) mg/dl and 8.7±6.0 (4.2 −21.5) µU/ml).In line with the comparable glucose and insulin levels, the HOMA-IR was not different between the control group and the hyaluronidase treated group, 2.0±0.4 versus 2.4±1.8, respectively.

### 

#### 1) Glycocalyx Barrier Properties

For assessment of glycocalyx barrier properties, the microcirculation of the gastrocnemius muscle was visualized with a SDF camera. Dynamic variations in red blood cell (RBC) column width were measured in each visible useful microvessel and the position of the RBC-glycocalyx interface was used to monitor the level of penetration of RBCs into the glycocalyx (Fig. 1). In the control rats, the coefficient of variation between the 10 measurements in a vessel during the baseline period for D_P50_ and D_perf_ were 4.5±2.4% and 6.4±5.0% respectively; pooled values were respectively 9.5±1.3 µm (D_P50_) and 13.4±1.2 µm (D_perf_). Mean coefficients of variation of D_P50_ and D_perf_ within an animal were 11.3±3.0% and 15.0±5.5%, between animals they were 3.5% and 9.0% and between vessels they were 12.4% and 18.6%, respectively. Subsequent insulin infusion resulted in an increase in outward movement of the RBC-glycocalyx interface of 0.34±0.44 µm at both sides within 10 minutes (p<0.05; Fig. 1C, right panel), while there was no significant effect of insulin on RBC-glycocalyx interface when tested for the entire 30 minutes infusion period. D_P50_ did not change within the first 10 minutes after start of the insulin infusion, nor during the 30 minutes of insulin infusion (Fig. 1C, left panel). Compared to the control rats, D_P50_ in the microvessels of the hyaluronidase treated rats was lower during baseline (8.6±0.9 µm; p<0.05) and did not change in response to insulin infusion as well (Fig. 1D, left panel). The D_perf_ at baseline did not differ (12.6±1.4 µm) in the hyaluronidase treated rats compared to the control rats; in contrast to control, D_perf_ did not increase in the initial 10 minutes after start of insulin infusion in the hyaluronidase treated rats (Fig. 1D, right panel). The response of D_perf_ during the insulin infusion was heterogeneous with respect to the time of the peak and the magnitude of the response between vessels within an animal. When statistical analysis was performed on the total number of vessels, D_perf_ was found to be increased in control animals at t = 5, 10, 15, 20 and 30 minutes after insulin infusion compared to baseline, without a change in D_P50._ In contrast, both D_perf_ and D_p50_ were unaffected at any time point after insulin infusion in the hyaluronidase treated animals.

**1a) Capillary tube hematocrit.** Rapid changes in capillary hemodynamics in response to hyperinsulinemia were evaluated in cremaster muscle with intravital microscopy. Capillary tube hematocrit was derived from measurements of capillary RBC flux, velocity, and anatomic diameter, in multiple vessels in an experiment (see Methods). These parameters showed a considerable range during baseline conditions already, resulting in a mean coefficient of variation of H_cap_ within an animal of 51.8±16.2%, between animals of 20.2%, and between vessels of 47.8% in control rats. Nevertheless, capillary tube hematocrit was observed to significantly increase within the first 10 minutes after start of the insulin infusion already (Table 3). RBC flux tended to increase (p = 0.08), without changes in RBC velocity and anatomic diameter. Hyaluronidase treatment itself was associated with an elevated capillary tube hematocrit under baseline conditions, which was not further increased by insulin infusion within the first 10 minutes (Table 3). However, RBC flux tended to decrease after insulin infusion in the hyaluronidase treated rats (P = 0.06).

**Table 3:** Capillary hemodynamics measured in the cremaster muscle.

	CONTROL		HYALURONIDASE	
	Baseline (n = 13)	Insulin 10 min (n = 14)	Baseline (n = 9)	Insulin 10 min (n = 7)
**Hcap (%)**	21.1±10.1	29.0±9.8*	42.6±20.2 #	40.6±25.9
**RBC velocity (µm/s)**	216±93	207±83	220±133	148±89
**RBC flux (cells/s)**	18.2±11.8	27.6±15.1	23.5±13.0	12.9±5.6
**Anatomic diameter (µm)**	6.2±1.5 (4.6–10.0)	6.5±1.4 (4.9–9.0)	5.4±0.55 (4.9–6.5)	5.0±0.7 (4.0–5.7)
**Capillary blood flow (pL/s)**	6.3±3.4 (1.6–16.1)	6.7±2.8 (3.2–11.9)	5.0±3.3 (0.8–10.3)	2.9±1.9 (0.7–6.2)

Capillary hemodynamics were obtained during baseline conditions and in the first 10 minutes period during infusion of insulin in control (saline-treated) rats (n = 3) and hyaluronidase-treated rats (n = 2). Data are means ± SD, number of vessels given in upper row.

* P<0.05 effects of insulin compared to baseline (unpaired student’s t-test),

# P<0.05, hyaluronidase baseline compared to baseline control rats (unpaired student’s t-test).

#### 2) Insulin-mediated Glucose Disposal

**Figure 2:**
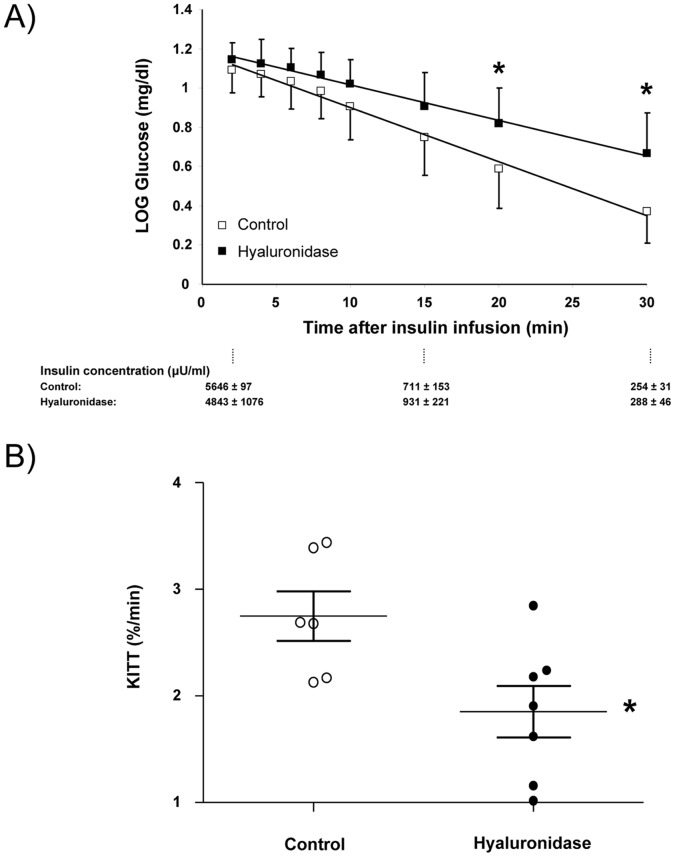
Mean ± SEM of the logarithm of blood glucose in time after an i.v. bolus of insulin (1 U/kg) in control rats (n = 6) and hyaluronidase treated rats (n = 7) are shown in panel A. Insulin levels (in µU/ml) measured at different times during the test are shown in the panel below the graph.*, P<0.05 versus control (unpaired student’s t-test). The glucose disposal rate (Kitt), calculated from the slope of the linear regression line of the logarithm of blood glucose against time multiplied by −100, for each individual experiment as well as the mean ± SEM of all experiments are shown in panel B *, P<0.05 versus control (unpaired student’s t-test).

To determine the effect of glycocalyx degradation on whole-body insulin-mediated glucose disposal, IVITTs were performed in anesthetized rats, and the glucose disposal rate monitored for 30 min. The Kitt was decreased in the hyaluronidase treated rats compared to control rats (p<0.05) (Fig. 2). Systemic hematocrit before the infusion of glucose was 43.8±3.4% in the control rats and 46.3±7.4% in the hyaluronidase treated rats and did not change after the insulin infusion in both groups of animals.

**Figure 3:**
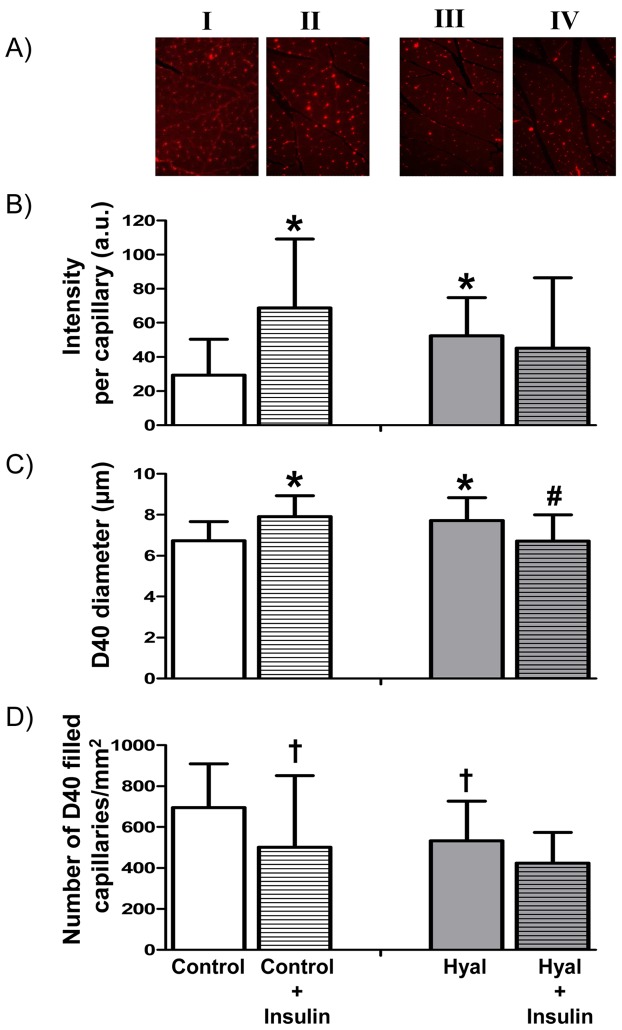
Typical examples of histological cross sections of rat hindlimb muscles at baseline in control rats (1 hour after saline; I, n = 18 slides), 30 minutes after insulin administration in control rats (II, n = 13 slides), 1 hour after a bolus of hyaluronidase (III, n = 17 slides) and 30 minutes after insulin in hyaluronidase treated rats (IV, n = 11 slides) (**A**)**.** In each slide the total intensity (B) and diameter (C) of each tracer filled capillary were determined, as well as the number of filled capillaries (D) (see Methods). In muscles of rats in which no tracer was infused (n = 12), the number of counted capillaries was 102 and total intensity in these capillaries was 18.8; data in panels B and D are corrected for these values. *, P<0.05 versus control baseline (unpaired student’s t-test); ^†^_,_ P<0.05 versus control baseline (Mann-Whitney U test);^ #^_,_ P<0.05 versus hyaluronidase baseline (unpaired student’s t-test).

**2a) D40 distribution.** Figure 3A shows representative histological images of D40 filling in capillaries of hindlimb muscle in the four different treatment groups. These images were used to determine both intensity (Fig. 3B) and diameter (Fig. 3C) for each filled capillary, as well as the total number of D40 perfused capillaries per muscle area (Fig. 3D). Mean intensity and number of fluorescent capillaries were 18.8±7.1 a.u. and 102±62 capillaries/mm^2^ respectively, in muscle without D40 infusion; these autofluorescence values were subtracted from the experimental values presented in Figure 3. Both mean intensity, as well as diameter of the D40 filled vessels were greater (p<0.05) in the rats that underwent the IVITT compared to the rats that did not receive insulin. The number of D40 filled capillaries was lower in the rats that received insulin (p<0.05, Fig. 3D).

In the hyaluronidase treated rats, mean intensity and D40 diameter were greater compared to control baseline (P<0.05), and the number of D40 filled capillaries was significantly lower. In contrast to the effect of insulin in control animals, mean D40 intensity did not increase and the diameter of D40 filled capillaries was actually reduced (p<0.05) at the end of the IVITT in the hyaluronidase treated rats. In these rats, insulin infusion appeared not to affect the number of D40 filled capillaries. Structural capillary densities (WGA-FITC staining) were not different in the four groups (control: baseline 821±206 capillaries/mm^2^, insulin 816±120 capillaries/mm^2^; hyaluronidase: baseline 989±198 capillaries/mm^2^, insulin 766±189 capillaries/mm^2^).

## Discussion

The observations in the current study demonstrate that insulin rapidly increases the accessibility of circulating blood into the glycocalyx, which enables an increase in blood volume within already perfused capillaries. In contrast, after enzymatic glycocalyx degradation the ability of insulin to modulate glycocalyx properties and to increase capillary blood volume in individual capillaries is impaired, and this impairment is associated with a ∼35% reduction in insulin-mediated glucose disposal from the circulation. While confirming previous studies indicating that the ability of insulin to dispose glucose is coupled to its ability to increase capillary blood volume in muscle [5], [6], the current results indicate that the latter includes volume increases at the level of the individual capillary by insulin increasing the accessibility of the glycocalyx for flowing blood.

### Methodological Considerations

#### SDF imaging

The microcirculation on the external part of the gastrocnemius muscle was monitored with SDF imaging. The advantage of the SDF camera is that it can readily be applied for visualization of the sublingual microcirculation in humans [20], [27], [28], [29], and of skeletal muscle microcirculation in rodents as done in the current study. Changes in glycocalyx barrier properties were assessed using specialized software tools developed in our laboratory. Since the anatomic borders of the vessels are not depicted when using epi-illumination, we analyzed the dynamic range of the RBC column width as a reflection of the glycocalyx barrier properties (Fig. 1B). The analysis requires sufficient continuous RBC filling of the microvessels. The average D_p50_ was ∼9 µm, illustrating that the analysis was not directed to true capillaries only and most likely included pre- and post-capillary microvessels. SDF measurements were reproducible during the baseline period in control rats as indicated by the small coefficients of variation for D_p50_ and D_perf._ The SDF camera has a low magnification, giving pixel sizes of 1.26 µm. Linear interpolation of the intensity profiles into virtual subpixels of ∼0.15 µm, however, allowed for more accurate measurements of FWHM positions while in addition to this, there were 21 measurements along the length of a vascular segment for each measurement site. The analysis revealed that during control conditions, D_P50_ was bordered by a cell poor region of approximately 2 µm at both sides. This region resembles the so-called perfused boundary region (PBR; Fig. 1), which results from the phase separation between the RBCs and plasma in microvessels and includes the most luminal part of glycocalyx that allows cell penetration [30], [31], [32]. Accordingly, an increase in D_perf_ at a given D_P50_ width is indicated to reflect an increased glycocalyx accessibility due to impaired glycocalyx barrier properties (as indicated by the arrow in Fig. 1B) [23]. In line with this approach, Vink et al. recently showed, using high magnification bright-field microscopy in microvessels of the cremaster muscle of transgenic mice in which the endothelial cells were labeled with a green fluorescent protein, that hyaluronidase treatment was associated with an outward radial displacement of RBCs without a change in anatomic vessel diameter [33].

#### Intravital microscopy

Agonist- or enzyme-induced impairments of glycocalyx barrier properties have been shown to coincide with increases in functionally perfused capillary volume, as reflected by an increased capillary tube hematocrit [10], [14], [21]. To confirm that the increases in glycocalyx accessibility in the microvessels during insulin infusion would be accompanied by increases in capillary blood volume, we performed additional intravital microscopy experiments in a small number of animals, in which capillary hemodynamics were measured in multiple vessels in one animal [10], [21]. Capillary tube hematocrit was derived from RBC flux and velocity, anatomic diameter and MCV; while these measurements have potential errors associated with them this estimate has been used in many previous studies and the possible error associated with the calculation is regarded to be small [34]. In line with previous studies [10], [35], [36], capillary tube hematocrit showed a considerable range, as illustrated by a coefficient of variation between vessels of 48%; nevertheless, in line with our previous study [10], we observed a rapid and substantial increase in this parameter upon insulin infusion. Compared to the increases in capillary tube hematocrit of ∼250% found after bradykinin and SNP administration [10], the increases in hematocrit found with insulin were, however, relatively small, indicating that there still seems potential for capillary blood volume to increase further. Of note, MCV was based on previous studies [24], [25]; while effects of insulin or hyaluronidase on MCV have not been reported, possible affects of a change in MCV on capillary tube hematocrit in the present study cannot be ruled out.

#### IVITT

In accordance with previous studies [37], [38] we used an IVITT for measuring insulin sensitivity. We used an IVITT instead of a clamp as a more physiological stimulus with respect to the timing of the vascular insulin effect. When using a hyperinsulinemic euglycemic clamp, the glucose infusion rate is typically measured during steady state conditions which occur approximately 120 minutes after start of insulin infusion and it might be anticipated that at this time point insulin-mediated glucose uptake is dominated by processes occurring at the level of the myocytes and not at the level of the microvasculature. Note that in order to avoid hypoglycemia and to have a useful range for blood glucose to be decreased by insulin, we first gave a bolus of glucose in our IVITT experiments. As a result of the glucose bolus, counterregulatory mechanisms kicked in later than 30 minutes in our experiments, as shown by the dynamics of the glucose response (Fig. 2A), thereby warranting use of the entire blood sampling period for calculation of the Kitt.

#### Histology

To check if insulin-mediated increases in capillary blood volume occurred during the IVITT, fluorescent D40 was infused as a small-sized plasma tracer [15] at the end of the test (30 min), and its distribution in the hindlimb muscle microcirculation was analyzed on histological sections. While during control conditions approximately 80% of the present capillaries (i.e. labeled by WGA) were filled with D40, the number of D40 filled capillaries decreased after hyaluronidase and insulin infusion. The lower number of D40 filled capillaries may reflect a redistribution of blood flow into the capillary network, as was previously described for hyaluronidase by Cabrales et al. [18], as well as an increase in the number of capillaries containing an erythrocyte (and hence no D40) in the histological cross-section. Further, the increases in D40 diameter occurred without changes in systemic hematocrit suggesting that the increase in D40 intensity did not reflect an increased D40 concentration in the plasma due to fluid leakage. Although all D40 filled microvessels were used for the analysis, capillaries were prominent in these cross-sections, as reflected by the calculated D40 diameters (Fig. 3C).

#### Hyaluronidase treatment

The rationale for using hyaluronidase treatment to study the role of the glycocalyx in insulin-mediated responses comes from previous studies which demonstrated loss of glycocalyx structures [39] and impairment of its barrier properties after administration of the enzyme [18], [19], [40]. These studies indicated that a period of ∼45–60 minutes was needed for an optimal effect of hyaluronidase; hereafter, the effect waned, but was still present after 2 hours [19]. Based on these findings we used an incubation period of 1–1.5 hour for the enzyme in the current experiments. In line with these previous studies, hyaluronidase treats rats showed decreased glycocalyx barrier properties which coincided with increases in capillary blood volume. Thus, hyaluronidase treatment was associated with a significant increased D_perf_ compared to the D_P50_ and increased capillary tube hematocrits in the cremaster microcirculation at baseline; furthermore, in the histology experiments, the intensity of D40 within a capillary, as well as D40 diameter were higher in the hyaluronidase treated animals compared to controls at baseline This observation appears to contrast the finding of a smaller D_P50_ in the SDF experiments. However, when taking into consideration that the vessels examined with SDF were not primarily capillaries but included also small arterioles and venules, it is possible that increased blood volume in the capillaries is associated with a reduced blood volume in the upstream and downstream microvessels. In line herewith, a previous study of VanTeeffelen et al [40] showed that hyaluronidase treatment was associated with decreases in anatomic diameter in second- and third-order arterioles in the mouse cremaster, which was suggested to reflect a decreased NO-bioavailability.

### Insulin-mediated Blood Volume Increase within Already Perfused Capillaries by Rapid Modulation of Glycocalyx Barrier Properties

In previous studies, using contrast-enhanced ultrasound (CEU) and 1-methylxanthine metabolism (1-MX), it has been shown that insulin infusion results in a rapid increase in microvascular blood volume and functionally available endothelial surface area in skeletal muscle [5], [41], [42], [43]. Based on these studies, the concept was put forward that these microvascular effects of insulin may promote its own delivery and that of glucose towards the myocytes [5], [42], [44]. These previous studies did, however, not distinguish whether the increase in capillary volume or surface area were the result of an increased number of perfused capillaries, an increased volume in already perfused capillaries, or both. Based on the idea that skeletal muscle capillaries at rest are only partly perfused [45], the data so far have been merely interpreted as insulin increasing the number of perfused capillaries by inducing vasodilatation of precapillary arterioles [46], [47], [48]. The data of the current study show that insulin, by attenuating glycocalyx barrier properties, may increase capillary blood volume within already perfused capillaries. As reflected by the outward movement of the RBC-glycocalyx interface, insulin stimulated farther penetration of the outer RBC edge into the glycocalyx by 0.34±0.44 µm within 10 minutes, indicating that the barrier properties of the glycocalyx were rapidly decreased by insulin. Reports by others showed that the dose of insulin used in the current study increases total skeletal muscle blood flow only after 20 to 30 minutes [47], [49], confirming that the agonist-induced impairment of glycocalyx barrier properties might precede its action on the resistance vasculature [21]. Capillary tube hematocrit in cremaster muscle increased with ∼40% within 10 min after start of a continuous insulin infusion, and we observed a more than 2-fold increase in the amount of D40 per capillary in hindlimb muscle capillaries 30 min after a bolus injection of an even higher dose of insulin. The D40 increase in control rats was partially due to an increase in D40 diameter, which is in line with the increased D_perf_ during insulin infusion found with SDF imaging. These results confirm previous studies which showed that insulin rapidly increases capillary blood volume in muscle tissue; our data show that the increase can occur within already perfused capillaries by an increase in glycocalyx accessibility. The effect of insulin on glycocalyx barrier properties and capillary blood volume was greatly impaired after enzymatic degradation of the glycocalyx with hyaluronidase (Fig. 1C, Table 3, Fig. 3B), which is in line with previous studies, which showed a lack of effect of agonists on glycocalyx dextran accessibility and capillary tube hematocrit after glycocalyx degradation [10], [17]. Subsequent insulin infusion in the hyaluronidase treated rats was even associated with smaller D40 diameters in the histological slides, which contrasts to the unaltered capillary diameters in the intravital microscopy experiments. The differences could be due to a difference in timing between the observations and/or the different insulin concentrations used. The decrease in D40 diameter might reflect a reduction in capillary anatomic diameter for example due to perivascular edema [39], or a reduction in capillary pressure coinciding with the impaired capillary perfusion observed in the IVM experiments, which could be a consequence of vasoconstriction of arterioles as also illustrated by the reduced median RBC column after hyaluronidase treatment.

### Glycocalyx Degradation is Associated with Impaired Vascular and Metabolic Insulin Actions

Hyaluronidase treatment was associated with similar changes in baseline glycocalyx properties and capillary blood volume as compared to the effect of insulin during control conditions, but apparently did not affect insulin sensitivity at baseline as illustrated by unchanged fasting glucose levels, fasting insulin levels and HOMA-IR compared to fasting control conditions. Importantly, insulin infusion in the hyaluronidase treated rat was accompanied by a ∼35% delayed glucose disposal from the circulation. It has been indicated, using 1-MX and CEU, that insulin increases capillary surface area for exchange by recruiting the number of perfused capillaries, and it is suggested that these increases in capillary blood volume are important for insulin’s ability to efficiently dispose glucose from the circulation [5], [6], [45]. However, the hyaluronidase results of the current study suggest that a single increase in capillary blood volume within individual capillaries in itself is not sufficient to increase glucose disposal. In the hyaluronidase treated animals, the decrease in insulin-mediated glucose disposal may be explained by an impaired microvascular delivery of insulin and glucose due to a compromised capillary perfusion, as evidenced by the reduced number of D40 filled capillaries, the decrease in RBC flux, and smaller D40 filled diameters found after insulin infusion in these animals.

The findings of the current study suggest that an intact glycocalyx is necessary for optimal regulation of transcapillary transport of insulin. It has been shown that the transcapillary exchange of insulin, despite its small size (6 kDa), does not simply occur via diffusion but that it likely involves a receptor-mediated process, involving caveolae [50]. Further, Vink & Duling [15] showed that the penetration of plasma proteins/peptides into the glycocalyx most likely involves molecular interactions with low affinity glycocalyx binding sites and does not simply follow a pattern based on molecular size or charge alone. It is therefore hypothesized that glycocalyx components like heparan sulfate proteoglycans may interact with insulin and affect its transcapillary transport. Numerous studies have reported that these polysaccharide structures are able to store various plasma proteins, to organize and regulate cell surface receptors, to interact with caveolae, and to be able to modulate the vasodilator effects of endothelial receptor-dependent, NO-mediated, agonists [51], [52]. In addition, a remarkable correlation between the localization of the insulin receptor and the presence of glycocalyx coating has been demonstrated in isolated adipocytes [53], and it might well be that this association also holds for the endothelium. The impaired insulin response after enzymatic glycocalyx modification might thus include compromised transport of insulin through the glycocalyx mesh towards its receptors on the endothelium.

In conclusion, the present study provides evidence that the endothelial glycocalyx plays a role in regulation of an efficient insulin and associated glucose delivery into skeletal muscle. The precise molecular and cellular mechanisms involved in the insulin-mediated modulation of glycocalyx barrier properties are unknown at the moment, and future studies are needed to determine the effects of insulin on the composition of the glycocalyx and to study the role of the glycocalyx in the transport of insulin towards and across the endothelium.

### Clinical Relevance

In recent years, it has been recognized that the endothelial glycocalyx exerts protective effects towards the vessel wall, in particular towards preservation of endothelial function. This layer was shown to be highly vulnerable to acute and chronic atherogenic, hyperglycemic and inflammatory conditions [13], [54], [55], and as a result, loss of the glycocalyx has been indicated a marker of, and potentially a contributing factor to, the development of endothelial dysfunction associated with conditions of increased cardiovascular risk. Since the current data show that glycocalyx degradation may not only be a consequence of hyperglycemia [56], [57], but that it may also directly compromise insulin-mediated uptake of glucose from the circulation by impairing the insulin-mediated capillary blood volume increase in muscle, loss of glycocalyx may be a common factor in the established relationship between insulin resistance and endothelial dysfunction in patients and experimental animals [58], [59]. As a result the glycocalyx may constitute an important target for early diagnosis as well as therapy in people at risk for the development of endothelial dysfunction and insulin resistance, such as in the metabolic syndrome. In this respect, a previous study showed that 8 week treatment with sulodexide, a mixture of the glycocalyx components heparan and chondroitin sulfates, improved both sublingual glycocalyx dimensions as well as the vascular retention of albumin in type II diabetes patients [27]. While the effect of sulodexide on insulin sensitivity was not determined in this latter study, our current data would suggest a potential improvement by this glycocalyx therapy as well.
